# *In vitro* enteroid-derived three-dimensional tissue model of human small intestinal epithelium with innate immune responses

**DOI:** 10.1371/journal.pone.0187880

**Published:** 2017-11-29

**Authors:** Ying Chen, Wenda Zhou, Terrence Roh, Mary K. Estes, David L. Kaplan

**Affiliations:** 1 Department of Biomedical Engineering, Tufts University, Medford, MA, United States of America; 2 Department of Molecular Virology and Microbiology, Baylor College of Medicine, Houston, TX, United States of America; 3 Department of Medicine, Baylor College of Medicine, Houston, TX, United States of America; National Cancer Institute, UNITED STATES

## Abstract

There is a need for functional *in vitro* 3D human intestine systems that can bridge the gap between conventional cell culture studies and human trials. The successful engineering *in vitro* of human intestinal tissues relies on the use of the appropriate cell sources, biomimetic scaffolds, and 3D culture conditions to support vital organ functions. We previously established a compartmentalized scaffold consisting of a hollow space within a porous bulk matrix, in which a functional and physiologically relevant intestinal epithelium system was generated using intestinal cell lines. In this study, we adopt the 3D scaffold system for the cultivation of stem cell-derived human small intestinal enteriods (HIEs) to engineer an *in vitro* 3D model of a nonstransformed human small intestinal epithelium. Characterization of tissue properties revealed a mature HIE-derived epithelium displaying four major terminally differentiated epithelial cell types (enterocytes, Goblet cells, Paneth cells, enteroendocrine cells), with tight junction formation, microvilli polarization, digestive enzyme secretion, and low oxygen tension in the lumen. Moreover, the tissue model demonstrates significant antibacterial responses to *E*. *coli* infection, as evidenced by the significant upregulation of genes involved in the innate immune response. Importantly, many of these genes are activated in human patients with inflammatory bowel disease (IBD), implicating the potential application of the 3D stem-cell derived epithelium for the *in vitro* study of host-microbe-pathogen interplay and IBD pathogenesis.

## Introduction

Studies on human intestine have gained increasing interest due to its vital role as the “second brain” in the human body[1]. The human small intestine is a highly complex hollow organ located at the upper part of the intestinal tract. It is comprised of an intestinal epithelium, lamina propria, submucosa, muscularis mucosa, and serosa. The small intestinal epithelium is the innermost layer featuring two topographic structures, the villi (luminal protrusions) and crypts (luminal invaginations), on the top of which trillions of commensal microbes reside[2]. The epithelium covering the villi encompasses at least four major cell populations: absorptive enterocyte cells, mucus-producing Goblet cells, hormone-secreting enteroendocrine cells (EECs), and antimicrobial peptide secreting Paneth cells in the crypt[3]. All intestinal epithelial cell types are derived from proliferative crypt regions containing undifferentiated intestinal stem cells (ISCs) that self-renew to maintain stem cell populations which are identified by the specific expression of leucine rich repeat containing G protein-coupled receptor 5 gene (Lgr5) [4]. The differentiated epithelial cells enable the small intestine to perform two major physiological functions: efficient absorbance of nutrients and water from ingested food and establishment of a dynamic physical and biochemical barrier against external toxins and invading enteric pathogens. Loss of either of these functions is associated with the initiation and propagation of several intestine diseases, such as bacterial, viral, and parasitic infections, and inflammatory bowel diseases, which affect millions of people worldwide[5, 6]. To develop effective solutions to this worldwide problem, animal models are utilized for studies related to its causes and treatments, however, costly facilities and lack of correlations to human physiological responses limit the relevance of these animal models. This disconnect has limited the development of effective treatments to combat many of these infectious diseases, leaving large populations around the world susceptible. Tissue engineering approaches offer an alternative strategy to recapitulate human intestinal structure and function *in vitro*, which circumvent the limitations of animal models and provide new experimental systems with which detailed study of disease and interventions can be pursued in a more effective manner[7].

In the past decade, there have been many attempts to recreate *in vitro* bioengineered intestine-like tissue models for the study of intestinal diseases and for the development of new therapies[8, 9]. Existing *in vitro* models of the human intestine rely on cultures of intestinal epithelial cell monolayers on cell culture platforms to mimic the human small intestine microenvironment. These culture platforms may be two-dimensional (2D) or three-dimensional (3D) and typically include flattened or ridged 2D substrates[10], microfabricated substrates[11], microfluidic chips[12–14], hollow fiber bioreactors[15], or biomaterial scaffolds[16–18]. The major pitfall of the abovementioned intestine models is the use of heterogeneous human colonic adenocarcinoma cell lines, such as Caco-2 and HT-29. Cell lines are not representative of native intestinal tissue in many ways. For instance, each cell line only comprises one single cell population and fails to recapitulate the cell diversity in normal intestinal epithelium. Furthermore, the genotype of the subclones of these cell lines, especially Caco-2 cells, tends to change with increasing passage numbers or with differing culture conditions, yielding at best, inconsistent drug screening and host-pathogen interaction data[19–22]. As a result, the pharmaceutical industry, which uses cell line-derived intestinal systems for drug testing purposes, suffers high attrition rates, with less than 10% of clinical drug candidates making it to phase I testing and entering the market[23]. To tackle the limitations of cell lines, tissue engineers have adopted primary human small intestinal epithelial cells (hInEpiCs) which are isolated directly from native intestinal tissues for the *in vitro* establishment of a more physiologically relevant human small intestinal epithelium[24, 25]. However, hInEpiCs are difficult to isolate, remain viable for only several days and readily lose their phenotype in culture, hampering their widespread application in tissue engineering. Therefore, an alternative non-transformed epithelial cell source is needed to model a physiological 3D human intestine.

Recent advances in human intestinal stem cell culture methods[26–29], particularly the isolation and the infinite expansion of crypt/stem cell-derived human small intestinal enteroids (HIEs), have provided a suitable source of nontransformed small intestinal cells. HIE cultures that contain LGR5-positive intestinal stem cells are generated *ex vivo* from small intestinal crypt samples (endoscopic biopsies or surgical tissues) of individuals consenting to tissue donation for research. HIEs grow in Matrigel as 3D spheroids capable of giving rise to all lineages of intestinal epithelium. Compared to cancer cell lines and hInEpiCs, HIEs have two main advantages. First, HIEs can self-renew, expand indefinitely and differentiate into all cell types of the intestinal epithelium. Secondly, HIEs are patient-specific, which may allow investigation of personalized therapeutics. Each enteorid has a micro-scaled enclosed lumen with apical cell surfaces facing the lumen and basal surfaces exposed to the Matrigel. For a long time, accessing the lumen of enteroids and inducing appropriate luminal stimulations or bacterial infections has been difficult, limiting their use in intestinal tissue modeling and disease studies. Recently, several groups have described approaches for flattening enteroids into monolayers to enable mechanistic studies of various bacterial and viral infections infections[30–32], highlighting the vast potential of HIEs as a valuable cell source for *in vitro* bioengineering of human small intestines and studying intestinal tissue development and disease.

It has been consistently observed that primary cells isolated from tissues will stretch out in an unnatural state and lose their phenotype when cultured on stiff 2D planar environment including tissue culture plastics [33, 34]. Conversely, some cell types cultured on 2D surfaces can restore their physiological morphologies and functions when seeded onto a 3D cell culture scaffold[35]. Thus, the development of novel 3D biomaterial scaffolds to promote tissue formation *in vitro* is of considerable importance. In an effort to develop 3D scaffolds for intestinal tissue engineering, our group previously bioengineered a novel 3D porous silk protein scaffold system with a hollow channel that can form a more physiologically relevant representation of the microenvironment for the residence of the intestinal epithelium derived from cell lines (Caco2/HT29-MTX) than currently available 2D systems[17].

In the present study, we adopted this 3D tubular silk sponge scaffold system for the *in vitro* cultivation of human intestinal stem cell-derived enteroid monolayers, extending the system beyond the use of immortalized cell lines as shown in the previous work. We prepared 3D scaffolds using silk fibroin which is an inexpensive and biocompatible biomaterial [36]. Primary human intestinal myofibroblast cells are dispersed in the porous scaffold bulk as feeder cells to improve the cell culture performance of the epithelium[37]. Caco-2/HT29-MTX and hInEpiCs seeded in the same scaffolds were used for the comparison of tissue structure and functions. The goal of this work was to assess nontransformed 3D human intestinal epithelium tissue models to bridge the gap between conventional cell culture studies and human trials, to achieve a greater understanding of intestinal biology and infectious disease mechanisms and to serve as a preclinical screening tool for pharmacological targets.

## Methods

### Cell cultures

*Human intestinal enteroid culture*–HIEs isolated from human jejunum were kindly provided by Dr. Mary Estes from Baylor College of Medicine through the Texas Medical Center Digestive Diseases Center Enteroid Core. The Baylor College of Medicine Institutional Review Board approved the study protocol (protocol numbers H-13793 and H-31910). Procedures for maintaining and passaging HIEs were previously described and included informed consent and written approval [38]. Briefly, frozen vials containing HIEs were thawed out and resuspended in Matrigel (25 μL/well, Corning). The Matrigel mixture was plated as droplets into 24-well tissue culture plates and incubated at 37°C for 5–10 minutes to polymerize the Matrigel. 500 μL of HIE growth medium, consisting of 15% Advanced DMEM/F12 (Invitrogen) supplemented with 100 U/ml penicillin-streptomycin (Invitrogen), 10 mM HEPES buffer (Invitrogen), and 1× GlutaMAX (Invitrogen); 10% Noggin-conditioned medium (made from Noggin-producing cells; kindly provided by G. R. van den Brink, Amsterdam, The Netherlands); 20% R-spondin-conditioned medium (R-spondin-producing cells; kindly provided by Calvin Kuo, Palo Alto, CA); 50% Wnt3A-conditioned medium produced from ATCC CRL-2647 cells (ATCC); 50 ng/ml epidermal growth factor (EGF) (Invitrogen), 10 mM nicotinamide (Sigma-Aldrich), 10 nM gastrin I (Sigma-Aldrich), 500 nM A-83-01 (Tocris Bioscience), 10 μM SB202190 (Sigma-Aldrich), 1× B27 supplement (Invitrogen), 1× N2 supplement (Invitrogen), and 1 mM N-acetylcysteine (Sigma-Aldrich), was added to each well. HIEs were used at passages 10–40. Live enteroids were imaged with a phase microscope (Leica). *Human Intestinal Myofibroblast cell culture–*H-InMyoFibs were purchased from Lonza and cultured in SMGM™-2 BulletKit™ medium (Lonza) according to the manufacturer’s instructions. Cells were used at the passages of 3–5. *Intestinal epithelial cell line culture***—**The Caco-2 (CRL-2102) cell line was obtained from ATCC, and HT29-MTX cell line was obtained from the Public Health England Culture Collections (Salisbury, Great Britain). Both Caco-2 and HT29-MTX cells were grown in DMEM supplemented with 10% fetal bovine serum, 10μg/mL human transferrin (Invitrogen), and 1% antibiotics and antimycotics (Invitrogen). For Caco-2 and HT29-MTX, cells from passage number 33–44 were used for the experiments. *Primary human intestinal epithelial cell culture***–**hInEpiCs were purchased from Cell Biologics (Chicago, IL) and cultured in complete epithelial cell medium (Cell Biologics) following the manufacturer’s protocol. Cell were used at passage 2. All cells were cultured in 37°C, 5% CO_2_ humidified atmosphere. The medium was changed every other day.

### Generation of 3D silk scaffolds

**Fig 1:**
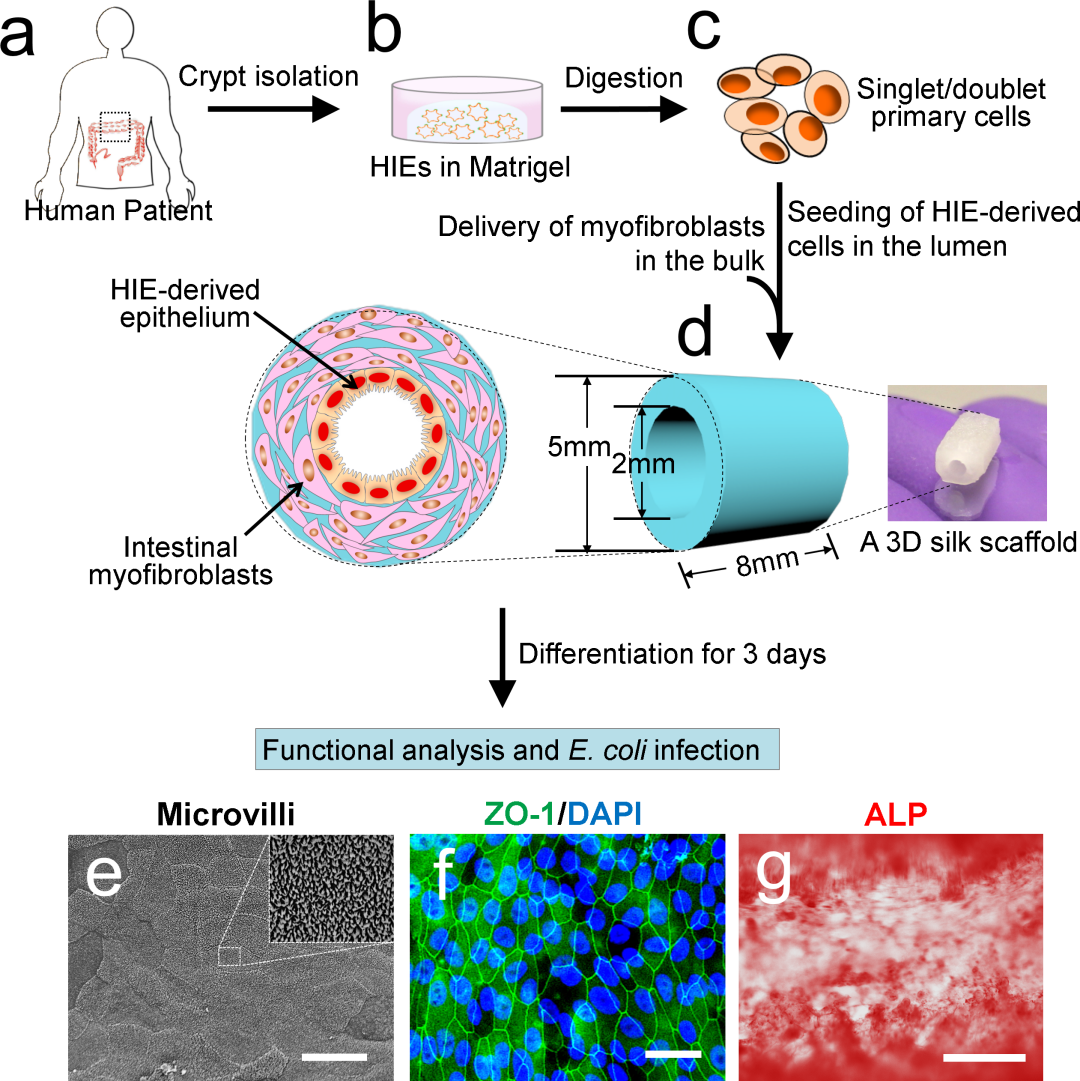
Overview of the cell seeding strategy for HIE-derived 3D intestinal constructs. (a, b) HIEs isolated from human patients are cultured in the Matrigel. (c) HIEs were enzymatically digested to obtain Singlet/doublet cells. (d) HIE-derived cells were seeded onto the luminal surface of a 3D tubular silk scaffold, while H-InMyoFibs were delivered into the spongy silk scaffold bulk. The constructs were cultured in differentiation medium for at least 3 days to induce intestinal epithelial differentiation. (e) SEM showed the microvilli brush border formation at the apical cell surface. Scale bar, 10μm. (f) Highly organized ZO-1 chicken wire pattern staining in differentiated HIE-derived epithelium on 3D scaffolds. Scale bar, 15μm. (g) ALP staining on the epithelial cells were observed on the epithelium. Scale bar, 250μm.

3D silk scaffolds were prepared as described previously and summarized in S1 Fig [17]. Briefly, silk fibroin was extracted from *Bombyx mori* silkworm cocoons. To prepare silk scaffolds with hollow channels, special cylindrical molds were cast from polydimethylsiloxane (PDMS; Down Corning). PDMS was prepared by mixing the base reagent with the curing reagent in a mass ratio of 10:1. The cylindrical PDMS molds consisted of a Teflon-coated stainless-steel wire (diameter, 2 mm; McMaster-Carr) inserted through the cross section of the cylinder to develop a hollow channel in the silk scaffold. Finally, a 4 to 5% (wt/vol) viscous silk solution was poured into the PDMS molds. The molds were frozen at −20°C overnight and then transferred to a lyophilizer for drying. The dried silk scaffolds were then autoclaved to induce the β-sheet conformations (insolubility in water), soaked in distilled water overnight, and trimmed along the axis of the hollow channel into a cuboid 5 by 5 by 8 mm. The fabrication method resulted in a scaffold consisting of a hollow channel space (diameter, 2 mm) and a bulk space around the channel that contained interconnected pores (Fig 1D).

### Cell seeding on 3D silk scaffolds

The general procedures for the seeding of intestinal epithelial cells and myofibroblast cells on 3D silk scaffolds were previously described[17]. The cell seeding strategy for HIE-derived constructs is summarized in Fig 1 and S2 Fig. For each HIE-derived scaffold, before being seeded on the scaffolds, 4 wells of HIEs were collected by washing with 500uL/well of 0.5mM EDTA (0.5M EDTA diluted 1:1000 in PBS), spun at 1200 rpm at 4°C for 5 mins. HIEs were then digested with 0.25% Trypsin for 4 mins at 37°C to obtain pellets containing singlets and doublets for luminal cell seeding. Pellets were resuspended in 35μL enteroid growth medium containing 10μM Y-27632 (Sigma-Aldrich), and then seeded onto each side of the scaffold lumen. Collagen gels containing 2×10^5^ H-InMyoFibs per ml were then delivered into the spongy silk scaffold bulks. HIE-derived scaffolds were first cultured in enteroid growth medium containing 10μM Y-27632 over night, and then switched to differentiation medium (growth medium without the addition of Wnt 3a, Nicotinamide and SB202190, and with 50% reductions in the concentrations of R-Spondin and Noggin conditioned medium). HIE-derived tissues were cultured in differentiation medium up to 14 days. For cell line-derived and primary cell-derived scaffolds, previously described procedures[17] for the cell seeding in each compartment of the scaffold were exactly followed.

### Immunofluorescence and confocal imaging

3D scaffolds with intestinal cells were fixed with 4% paraformaldehyde (PFA, Santa Cruz). Silk scaffolds were cut in half along the longitudinal axis to better expose the lumen to the blocking solutions and antibodies during the following incubation steps. All specimens were then permeabilized using 0.1% Triton X-100 in phosphate-buffered saline (PBS, Invitrogen), then blocked with 5% bovine serum albumin (BSA, Sigma-Aldrich) for 2 hours. These specimens were incubated overnight at 4°C with anti-human ZO-1(1:100, BD Transduction Laboratories), anti-human-SI (sucrase-isomaltase) (1:100, Santa Cruz Biotech), anti-human-Muc-2 (1:50, Santa Cruz), anti-human-Lyz (1:100, Lysozyme, Abcam) and anti-CghA (1:100, Chromogranin A, Abcam, 1:100), then immersed in Alexa Fluor 488 donkey anti-mouse and Alexa Fluor 546 goat-anti-rabbit secondary antibodies (Invitrogen) at a dilution of 1:250, respectively. Scaffolds were then counterstained with dihydrochloride (DAPI, Invitrogen) before being mounted using Vectashield mounting medium (Vector Laboratories). For live staining, calcein-AM (Invitrogen) was used at different time points, following manufacturer’s guidelines. These 3D scaffolds were scanned using a Leica SP2 confocal microscope (Leica Microsystems) and Nikon A1R (Nikon Instruments Inc.) with Z-series capability. Scaffolds were observed under a confocal microscope with a filter set for DAPI (Ex/Em: 350/470 nm), Texas Red (Ex/Em: 540/605 nm) and GFP/FITC (Ex/Em: 488/514 nm). Confocal maximum projection images were assembled with Leica confocal software (ver 2.61, Leica), NIS-Elements AR software package (ver 4.20.01, Nikon) and ImageJ.

### Alkaline phosphatase (ALP) stain

ALP staining was performed using the Vector Red Alkaline Phosphatase Substrate Kit I (Vector Laboratories) according to the manufacturer’s protocol. Briefly, transwells and silk scaffolds with cells were fixed with 4% PFA for 1 minute at room temperature, then washed two times with PBS. The specimens were incubated with substrate solution at room temperature until suitable staining developed, and were then imaged with the Olympus MVX10 macroscope and captured by CellSens Dimension (ver 1.8.1) program.

### Scanning Electron Microscopy (SEM) and quantification of microvilli

Silk scaffolds with cells were cross-linked with 2.5% glutaraldehyde (GA), followed by progressive dehydration in a graded series of ethanol (30%, 50%, 75%, 95% and twice in 100%, 30 minutes at each concentration). The samples were subsequently dried by critical point drying with a liquid CO2 dryer (AutoSamdri-815, Tousimis Research Corp.). Prior to imaging using a scanning electron microscope (Zeiss UltraPlus SEM or Zeiss Supra 55 VP SEM, Carl Zeiss SMT Inc.) at a voltage of 2~3 kV, the samples were coated with a thin layer (10 nm thick) of Pt/Pd using a sputter coater (208HR, Cressington Scientific Instruments Inc.).

### Measurement of oxygen profiles in vitro

The oxygen concentration profiles were measured using a PC-controlled Microx TX3 oxygen meter (PreSens Precision Sensing GmbH) equipped with a needle-type housing fiber-optic oxygen sensor (NTH-PSt1-L5-TF-NS40/0.8-OIW, 140 μm fiber tapered to a 50 μm tip). Prior to use, a two-point calibration was performed according to the manufacturer’s protocols with oxygen-free water (1% sodium sulfite, Sigma) corresponding to the 0% oxygen partial pressure and with air-saturated water corresponding to 100%. The needle probe was mounted on a custom-made micromanipulator capable of precisely positioning the measurement spot in the vertical direction. One complete turn of the screw knob resulted in 0.1 inch (2.5 mm) of travel. HIE-derived cells were cultured in 3D structures for 3 days post differentiation, hInEpiC-derived cells were cultured in 3D structures for 5 days post cell seeding, and cell line-derived cells were cultured in 3D structures for 15 days post cell seeding. Each of the 3D intestinal tissue scaffolds was then placed in an Eppendorf tube with its luminal direction oriented perpendicularly, and allowed to stabilize for 1 to 2 hours before taking measurements. In each step of probe advancement (0.05 inch/step), the oxygen tension reading was allowed to equilibrate for at least 5 minutes followed by data recording. At the end of each depth-profile measurement, the probe was retracted and the process was repeated 3 times for each sample. Five oxygen readings (30 sec/reading) were collected at each measurement position, subsequently averaged and plotted. To ensure the comparability between different samples, all three profiles were determined on the same day (within 6 hours) using the same probe and calibration.

### Co-culture of Escherichia coli (E. coli) on 3D silk scaffolds

The *E*. *coli* (BL23(DE3)), was used for infection experiments. Bacteria were grown overnight into stationary phase in 2xYT broth (LB, with 2x yeast extract and tryptone) at 37°C with rotation. The bacterial cells were harvested at the mid-log phase of growth (O.D.600 = 0.6) by centrifugation (3,000×g, 10 min, 4°C), washed with PBS and resuspended to an O.D.600 of 0.1 (~10^7^ cells/mL) in Lysogeny broth (LB) medium at 37°C with rotation overnight. Prior to bacterial inoculation, monolayers on scaffold lumens were washed with PBS, and cultured with fresh antibiotic-free medium supplemented with 5% inactivated fetal bovine serum for 24 hours. 3×10^7^ total CFUs were added to each scaffold.

### Quantitative RT-PCR

Intestinal epithelial cells on the luminal surface of scaffolds were detached with 0.25% trypsin-EDTA and a cell scraper. Total RNA was isolated using the Qiagen Mini mRNA Extraction kit. RNA was reverse-transcribed using High-Capacity cDNA Reverse Transcription Kit (Invitrogen, Carlsbad, CA) following the manufacturer’s instructions. Six nanograms of cDNA were used for real-time PCR amplification for each well, using primer sequences shown in S1 Table. For each gene tested we performed three experimental replicates and four biological replicates. Gene expression levels were normalized to the GAPDH mRNA level.

### PCR array for the antibacterial response genes

A human antibacterial response RT^2^ profile PCR array was performed as per the manufacturer’s instructions (Qiagen, Valencia, CA). Total RNA was extracted from uninfected and infected HIE-derived, hInEpiC-derived and cell line-derived scaffolds respectively. cDNA was prepared as mentioned in *Quantitative RT-PCR* section. The cDNA was mixed with RT^2^ qPCR master mix supplied by the manufacturer and real time PCR was performed in a 96-well plate format using Mx3000P qPCR System (Agilent Technologies, Santa Clara, CA). Data were analyzed using RT^2^ Profiler PCR Array Data Analysis Software version 3.5. β-actin gene was used for normalization.

### Statistical analysis

Data are presented as mean±SEM (n = 3–5). A two tailed t-test was performed to compare means between two groups, and Analysis of Variance (ANOVA) was performed to compare means of multiple groups. P-values≤0.05 were considered significant.

## Results

### The establishment of HIE-derived nontransformed intestinal epithelium

In the present study, we employed 3D hollow silk scaffold systems that our group previously developed for intestine engineering[17]. As previously reported, this silk-based scaffold system consists of a hollow channel space (diameter, 2 mm) and a bulk space around the channel containing interconnected pores (Fig 1D). We bioengineered the human intestine model by cultivating HIE-derived nontransformed epithelial cells on the luminal surface of silk scaffolds and primary human intestinal myofibroblasts (H-InMyoFibs) within the scaffold bulk space as feeder cells (Fig 1A–1D). After cell seeding, the HIE-derived scaffolds were maintained in growth medium overnight and then differentiation medium for up to 14 days. Three days after cell differentiation, the small intestinal epithelial cells derived from HIEs formed confluent monolayers on the luminal surface of 3D silk scaffolds. The process of differentiation led to the formation of brush border with well-developed microvilli (Fig 1E and S3 Fig), the presentence of apical ZO-1 tight junctions (Fig 1F), and a polarized distribution of membrane components, such as digestive enzymes, ALP (Fig 1G). Interestingly, the microvilli in HIE-derived epithelia are closely stretching parallel to each other, in contrast to the more random orientation of the microvilli in hInEpiC-derived and cell line-derived epithelia.

### Identification of four major epithelial cell populations

**Fig 2:**
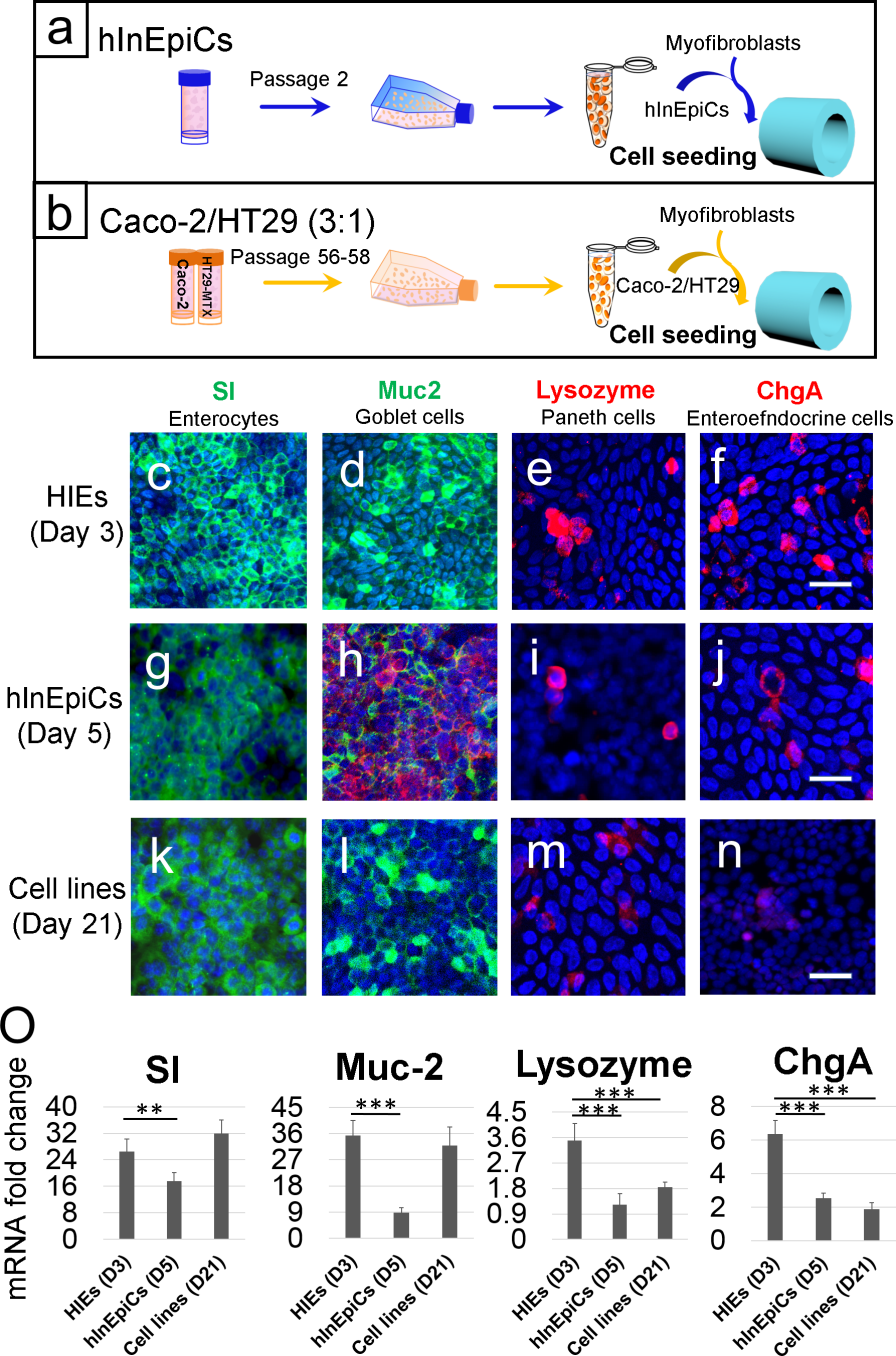
The differentiation of 3D intestinal epithelia. (a, b) General seeding cell seeding strategy for hInEpiC-derived and cell line-derived 3D intestinal constructs. (c-n) Immunohistological stainings of SI (sucrose-isomaltase, c, g, k), MUC-2 (Mucin 2, d, h, l), Lysozyme (e, i, m) and ChgA (Chromogranin A, f, g, n) showed the location of enterocytes, Goblet cells, Paneth cells, and enteroendocrine cells in differentiated HIE-derived, hInEpiC-derived cell line-derived epithelia. Scale bar, 25μm. (o) Relative mRNA expression levels of different markers of differentiated intestinal epithelia derived from the three different cell sources. The fold-change in mRNA expression was compared with cell line-derived 3D constructs at day 1 post cell seeding.

Native human small intestinal epithelium is populated with four major epithelial cell types: enterocytes, Goblet cells, enteroendocrine cells, and Paneth cells. Thus, we next aimed to identify the four cell populations from HIE-derived epithelium under differentiation on 3D silk scaffolds. Two other major cell sources for *in vitro* intestine engineering, Caco-2/HT29-MTX and hInEpiCs, seeded in the same scaffolds were used for the comparison (Fig 2A and 2B). Antibodies for each cell population were used for immunostaining. Using confocal microscopy, in HIE-derived epithelium, enterocytes were identified by Sucrase-isomaltase (SI) (Fig 2C), an enterocyte-specific, brush-border enzyme; Goblet cells by Mucin 2 (Muc2) (Fig 2D), a mucin exclusively and abundantly expressed by goblet cells; Paneth cells by Lysozyme (Fig 2E), specific marker for mature Paneth cells; and EECs by Chromogranin A (ChgA) (Fig 2F), a general cell surface marker for the enteroendocrine cells. Four markers were also observed in hInEpiC-derived epithelium (day 5 post cell seeding); however, the intensity of staining was relatively weaker. In cell line-derived epithelium (day 21 post cell seeding), the staining of Lysozyme and ChgA was not detectable, which means no Paneth cells and EECs present in the system. In addition to locating the protein expression of these markers by imaging, their gene expression in the epithelium grown in 3D constructs were also assessed by quantitative PCR. We found that differentiated HIE-derived epithelium (day 3) had the overall highest expression of these four marker transcripts than hInEpiC-derived (day 5) and cell line-derived epithelia (day 21) (Fig 2O).

### Intestine-specific gene expression analyses

**Fig 3:**
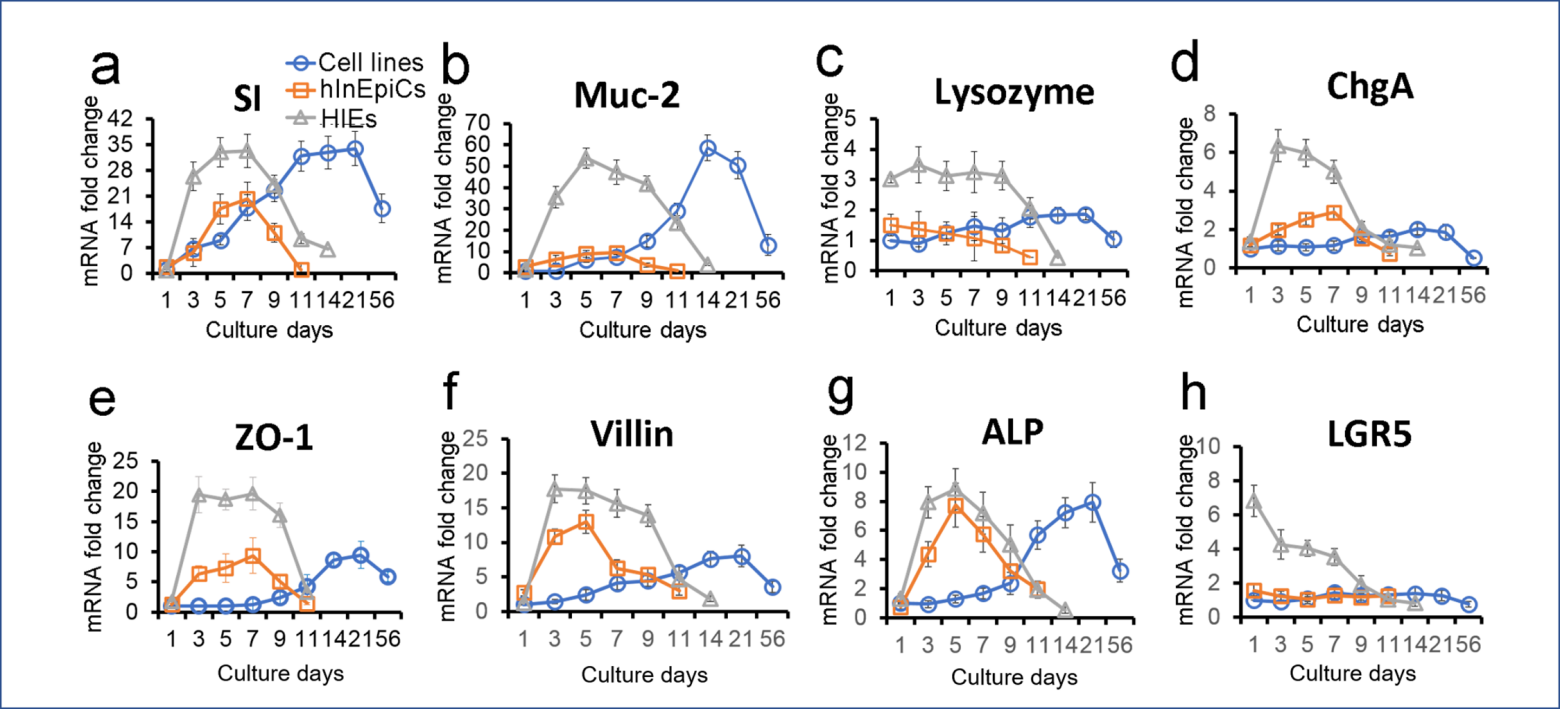
**Gene expression levels of four intestinal epithelial cell markers**, including SI (a), Muc2 (b), Lysozyme (c) and ChgA (d), functional epithelium markers, including ZO-1 (e), Villi (f), and ALP (g), and an intestinal stem cell marker, Lgr5 (h) were evaluated by quantitative reverse transcription-polymerase chain reaction (qRT-PCR) overtime in cultures. Data is presented as mean±SEM, n = 5 in each group, p<0.001. The fold-change in mRNA expression was compared with cell line-derived 3D constructs at day 1 post cell seeding.

Typically, the differentiation status of the cells is evaluated by transcript levels of representative characteristic markers. To further characterize HIE-derived epithelial cell types within the 3D scaffold culture, we performed mRNA expression analysis to directly quantify the gene expression levels of an extensive panel of known intestinal differentiation markers over time (Fig 3). The markers included the four abovementioned epithelial cell markers (SI, Muc2, Lysozyme and ChgA), mature epithelium markers (ZO-1, Villi and ALP), and an intestinal stem cell marker, Lgr5. HIE-derived epithelium showed a significant upregulation (~6–26 fold) of all marker transcripts after 3 days of cultivation in differentiation medium, with stable expression levels until around day 9 (Fig 3). In contrast, hInEpiC-derived constructs showed transient upregulated mRNA expression levels of SI, CghA, ZO-1, Villin, and ALP at day 5. The mRNA expression levels of the genes began to go down after day 7 (Fig 3A, 3D, 3E, 3F and 3G). Cell line-derived constructs achieved stable expression of marker genes between days 14–21. Although the enterocyte marker (SI) and Goblet cell marker (Muc-2) were highly expressed in cell line-derived epithelium on 3D scaffolds (Fig 3A and 3B), the Paneth cell marker (Lysozyme) and EEC marker (ChgA) were almost undetectable (Fig 3C and 3D). Generally, HIE-derived and hInEpiC-derived epithelia survived for shorter terms in culture (~9–12 days) than cell line-derived epithelium (~8 weeks); however, differentiated HIE-derived epithelium on 3D scaffolds reached maturity earlier (~3 days) than the hInEpiC-derived (~5–7 days) and the cell line-derived (~15–21) epithelia. Moreover, the overall expression levels of all markers from HIE-derived epithelium on 3D scaffolds were significantly higher than cell line-derived and hInEpiC-derived epithelia across all time points. The expression of Lgr5 transcript was only detectable in HIE-derived scaffolds and declined after differentiation (Fig 3H).

### Oxygen profiles in the scaffold lumens

**Fig 4:**
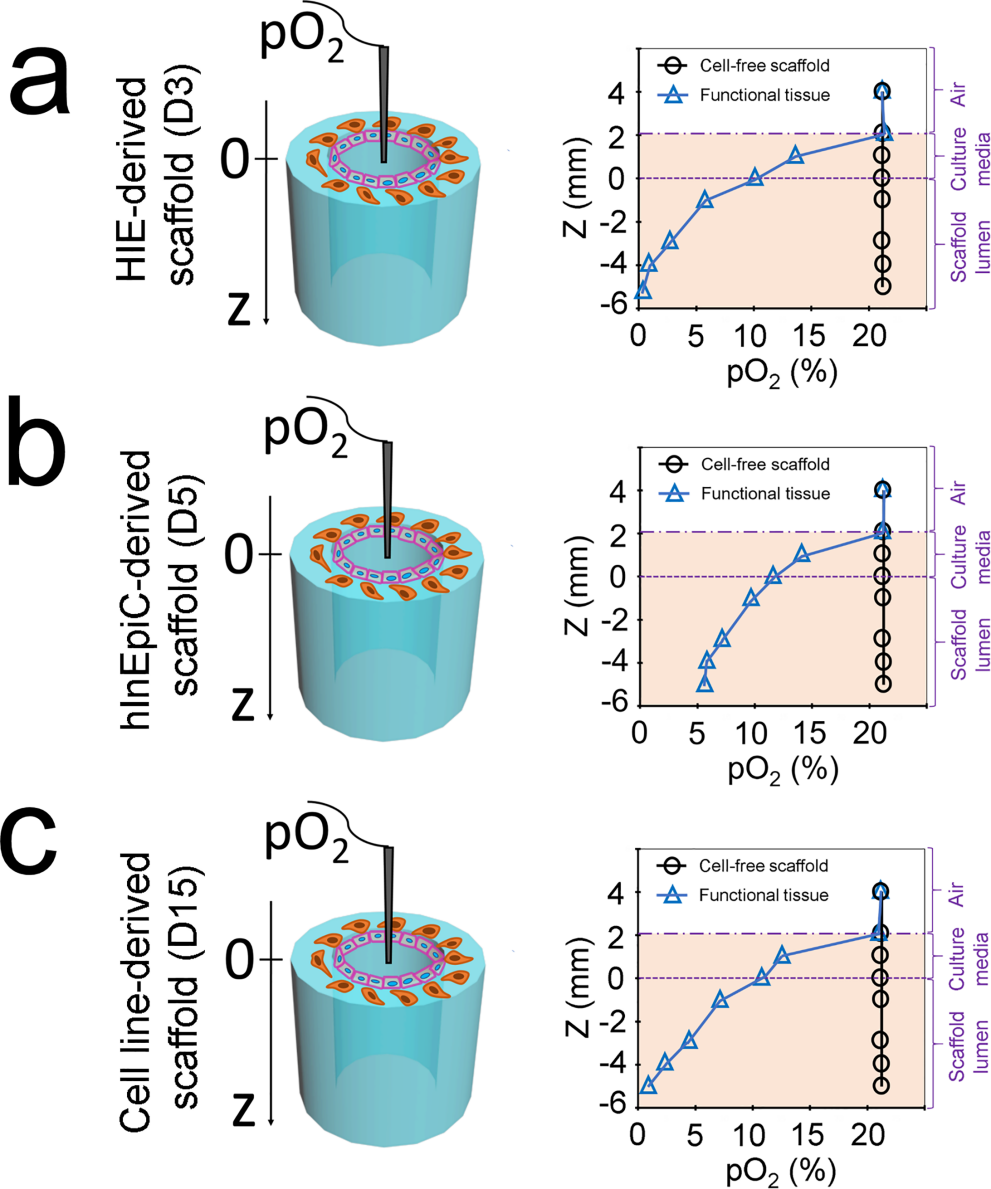
The oxygen concentration profiles of HIE-derived (a), hInEpiC-derive (b), and cell line-derived (c) tissues 3D tissues were measured using an oxygen meter.

An important feature of our 3D scaffold system is the hollow channel compartment for epithelial cell growth. This hollow structure enables decreased oxygen levels which mimic *in vivo* conditions through oxygen consumption kinetics and metabolic activities of the cells in the lumen[17]. In this study, we aimed to investigate whether HIE-derived cells grown in the lumen of the 3D scaffolds would also experience low oxygen tension. Similar to cell line-derived scaffolds (Fig 4C), HIE-derived scaffolds also exhibited depth-graded oxygen profiles in the luminal direction (Fig 4A). In the HIE-derived scaffolds, a region of microaerobic conditions (pO_2_ between 5% and 1%) was detected at depths ranging from 2 to 5 mm into the scaffold lumen; a nanaerobic region (pO_2_ ~1%) was detected at the depth of 5 to 6 mm. However, in hInEpiC-derived scaffolds, the lowest pO_2_ measured in the lumen was ~6% (Fig 4B).

### Antibacterial response to E. coli infection

**Fig 5:**
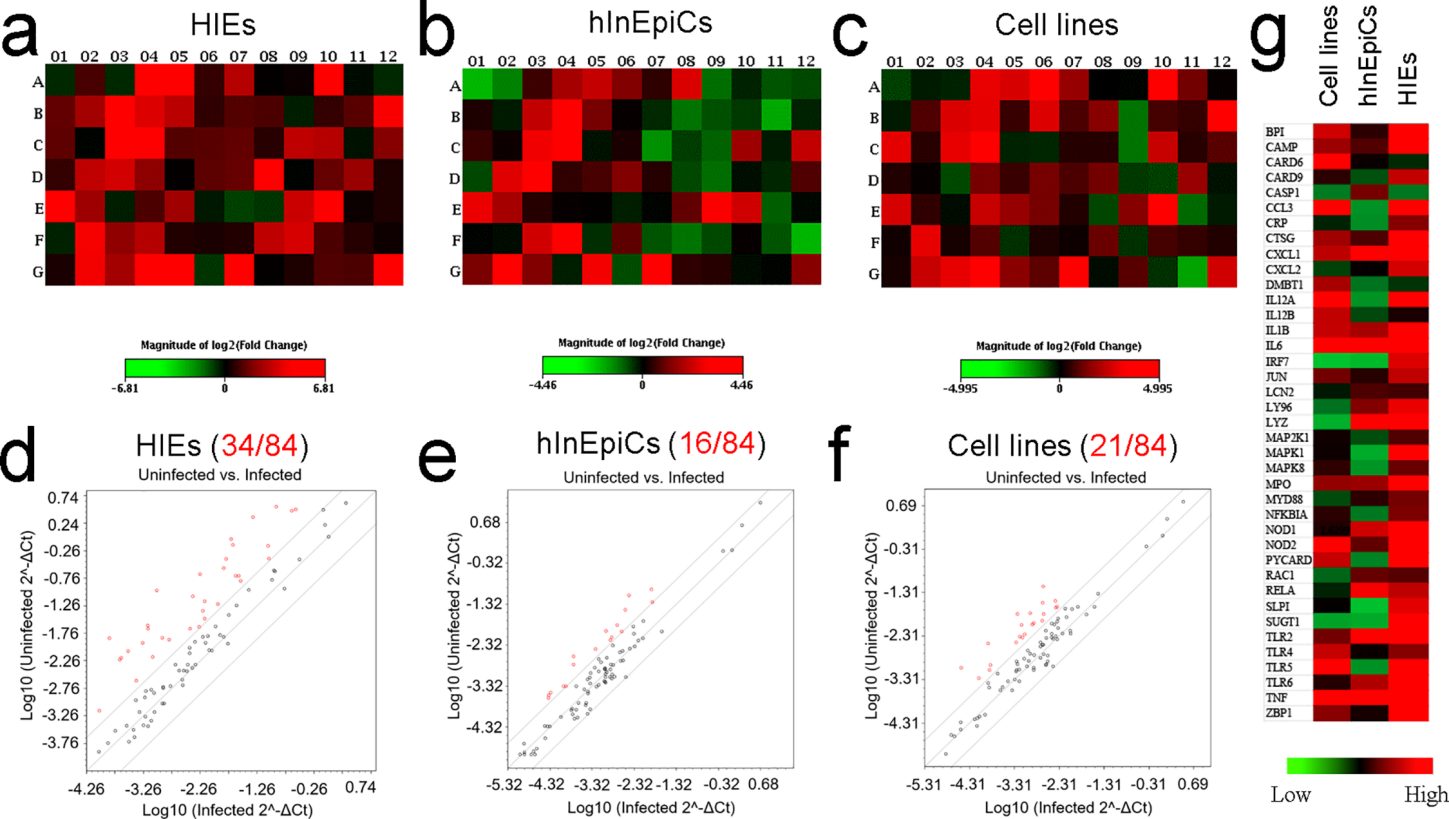
Human antibacterial response RT2 Profiler™ PCR arrays. (a-c) Heat-map comparison of 84 antibacterial genes in HIE-derived (a), hInEpiC-derived (b), and cell line-derived (c) epithelia after exposure to *E*. *coli*. for 4 hours. Genes were displayed for fold-change variation in respect to their uninfected control groups and colored by their normalized expression value (red: high expression; green: low expression). (d-f) Scatter plot charts. Genes upregulated with fold change greater than 4 and are showed as red dots; genes with fold change less than 4 are showed as green dots; unmodulated genes are showed as black. (g) Heatmap detail displayed all upregulated genes for HIE-derived, hInEpiC-derived, and cell line-derived epithelia after *E*. *coli* infection.

To determine the innate response of the intestinal epithelium against bacterial pathogens, we performed Human Antibacterial Response RT2 Profiler™ PCR arrays. Differentiated HIE-derived epithelium (3 days post differentiation), hInEpiC-derived epithelium (5 days in culture) and cell line-derived epithelium (15 days in culture) were separately incubated with a non-invasive strain of *E*. *coli* (BL23(DE3)). Gene expression profiles of epithelial cells from the three epithelial models were determined by the PCR array at 4 hours post inoculation, and compared with controls of each cell source without *E*. *coli* co-culture. The gene expression ratios between infected HIE-derived epithelium, infected hInEpiC-derived epithelium or infected cell line-derived epithelium and their corresponding uninfected control samples for all 84 genes were clustered and displayed in heat maps where individual elements of the plot are colored according to their standardized expression values (Fig 5A–5C; red squares: upregulated genes; green squares: downregulated genes). Compared to hInEpiC-derived and cell line-derived epithelia, HIE-derived epithelium displayed more upregulation after exposure to *E*. *coli* (more red squares). To more directly illustrate how these various source-derived epithelia on 3D scaffolds responded to the *E*. *coli* infection, the standardized expression values of all upregulated genes in Fig 5A–5C are displayed as a heat-map detailed in Fig 5G for the cell line derived, hInEpiC-derived and HIE-derived epithelium samples, respectively (Fig 5G; red squares: high expression; green squares: low expression). The dominant bright red color in HIE-derive epithelium indicated the enhanced antibacterial response of HIE-derived epithelium compared to hInEpiC-derived and cell line-derived epithelia. Based on gene selection criteria (P<0.05 and fold change≥4), we identified 34 upregulated genes for HIE-derived epithelium, 16 upregulated genes for hInEpiC-derived epithelium, and 21 upregulated genes for cell line-derived epithelium (Fig 5D–5F). Amongst all of the induced genes, microbial sensors/bacterial pattern recognition receptors (LY96, TLR2, TLR4, TLR5, TLR6, CRP, DMBT1, IRF7, ZBP1) and proinflammatory cytokines/chemokines (CCL3, CXCL1, CXCL2, IL12A, IL12B, IL1B, IL6) were predominant, followed by inflammatory mediator genes (MYD88, NOD1, NOD2, RAC1, RELA, TNF), antimicrobial genes (BPI, CAMP, CTSG, LYZ, MPO, SLPI), downstream signal transduction genes (MAP2K1, MAPK1, MAPK8, JUN, NKB1A), and some inflammasome signaling genes (CASP1, PYCARD).

## Discussion

The combination of stem cells and 3D biomaterial scaffolds has emerged as a promising strategy for tissue engineering[39]. Stem cell-derived enteroids from human patients have become a valuable *ex vivo* model of normal human intestinal epithelia, and this has become more obvious after recent advances in stem cell technology allowing the indefinite establishment and propagation from normal nontumorigenic human specimens. Our group previously developed a silk scaffolding system with a hollow architecture to support intestinal epithelial cells, and H-InMyoFibs under the intestinal epithelium. The 3D structure with the support of H-InMyoFibs enhances the differentiation of the intestinal epithelial cells and extends the cultivation of functional tissues compared to the 2D cell culture substrates [17]. In this study, we used stem cell-derived spherical HIEs from the intestinal crypt to investigate the possibility of growing the HIE-derived nontransformed epithelial cells in the 3D tubular silk scaffold system *in vitro* with H-InMyoFibs embedded in the system bulk for the tissue engineering of a 3D human intestinal epithelium. Our results suggest that the 3D silk scaffold system supports a functional intestinal epithelium derived from HIEs. The resulting epithelial tissues formed in the 3D scaffolds consisted of multiple differentiated and undifferentiated stem cells found in human native intestine, expressed elevated levels of transcripts of intestinal markers, generated low oxygen tension in the lumen, and demonstrated a significant anti-bacterial response to bacterial infections.

In this study, we adopted a previously published protocol[38] for the isolation and differentiation of single stem cells from patient-derived enteroids. As expected, the single cells formed a confluent monolayer on the luminal surface of the 3D scaffolds. Consistent with previous findings[31, 38], after culture in differentiation medium, four major native human intestinal epithelial cells were identified in the mature HIE-derived epithelium on 3D scaffolds by immunofluorescence analysis for protein expression (Fig 2C–2F) and qRT-PCR for expression of cell population-specific transcripts (Fig 3O). The cells included the absorptive enterocyte cells, the mucus-producing Goblet cells, the hormone-secreting EECs, and the antimicrobial peptide secreting Paneth cells. The epithelium also developed mature epithelial markers, including ZO-1 tight junctions, dense microvilli with brush border, and ALP production (Fig 1E–1G). Additionally, the gene expression of crypt stem cell marker Lgr5 was also detected in the tissue, indicating the existence of intestinal stem cells in culture (Fig 3H). These findings suggested that the 3D silk scaffold system provides an appropriate epithelial niche for the adhesion, proliferation and differentiation of intestinal stem cells. Silk protein as a scaffold has been fabricated to support wide variety of stem cells for different tissue engineering applications, such as cartilage[40], bone[41], adipose[42], etc. To our knowledge, this study is the first attempt at exploiting such systems for intestinal stem cell culture which permits cellular remodeling and tissue regeneration of a nontransformed human intestinal epithelium *in vitro*.

The marker gene expression is associated with the maturation of cells. Comparison of the gene expression of intestinal epithelial markers over time by qRT-PCR between HIE-derived, hInEpiC-derived and cell line-derived epithelia revealed distinctions between these cell lineages (Fig 3). In the three epithelia, the differentiated HIE-derived epithelium was characterized by the highest biological complexity with the highest expression of major marker transcripts. This is not surprising, as HIEs are of stem cell origin and are known to most closely resemble the native intestinal epithelium[28]. By contrast, cell lines are of cancer cell origin and can only represent one cell population of human intestine. Interestingly, though hInEpiCs are primary cells directly isolated from human tissues and express most intestinal epithelial markers, the overall gene expression levels of hInEpiC-derived epithelium were found to be lower than that of the HIE-derived epithelium. This result is in contrast with findings where marker expression of primary intestinal epithelial cells and intestinal stem cell-derived epithelial cells were on the same order of magnitude[25]. This inconsistency could be due to the source and passage of the primary cells used in the study. To obtain high enough cell numbers for scaffold seeding, we used cells from passage 2 after purchase from the vendor. Primary human cells tend to lose their genotype and cell viability with passage in culture. With this consideration, the lower expression of intestinal marker genes in the hInEpiC-derived epithelium could be explained by the loss of cell genotype during passaging. This may also explain the relatively higher oxygen tension (6%) that was detected in the lumens of hInEpiC-derived scaffolds. As proposed in our previous study [17], oxygen consumption of living cells in the lumen contributes significantly to the low oxygen state in the lumen. Cell activity and viability is correlated with oxygen consumption rate [43], and therefore, low epithelial cell viability of the hInEpiC-derived scaffolds reduced oxygen consumption and increased the oxygen concentration in the lumen. Notably, the expression of most marker genes in HIE-derived epithelium significantly declined beyond day 7 of culture, which recapitulates the *in vivo* life span of intestinal epithelial cells (5–7 days) [44]. These results indicate that human stem cell-derived enteroids may be the best choice for *in vitro* remodeling of human intestinal epithelium.

Low oxygen tension is critical for intestinal tissue function, as it is required for maintenance of a healthy gut microbial community[45]. *In vitro* generation and dynamic control of oxygen gradients mimicking *in vivo* intestinal tissue, which vary from the anaerobic lumen across the epithelium into the highly vascularized sub-epithelium, has been a challenge for bioengineering and tissue regeneration[46].

In an effort to overcome this challenge, we previously developed a 3D tubular silk scaffold system for intestine engineering in which cells are seeded on the luminal surface, which provides stable access to the full range of oxygen conditions but without exposure to a low oxygenated cultivation atmosphere. In this study, we take advantage of the luminal geometry to cultivate HIEs. Similar to cell line-derived intestinal models, the 3D *in vitro* HIE-derived intestinal models were also capable of reaching microaerobic, nanaerobic or anaerobic conditions in a standard CO_2_ incubator (21% O_2_, 5% CO_2_, 37°C) (Fig 4). Under physiological conditions, the intestinal mucosa experiences frequent and wide fluctuations in blood perfusion and metabolism. For example, the villus oxygen tension in the murine small intestine is reported as being ~2% under normal condition but decreases to ~0.5% during glucose absorption[47]. During acute or early stage gastrointestinal tract infections, pathogenic microorganisms and toxins, which enter the intestinal lumen and disrupt the mucous layer, trigger or exaggerate imbalances in tissue oxygen supply and demand. The bioengineered oxygen profiles and outcomes *in vitro* provide opportunities to study the role of oxygen concentrations in a wide variety of biological scenarios such as physiological stresses and pathological stimuli.

While the tissue characteristics of the HIE-derived epithelium on 3D scaffolds are very encouraging based on intestinal marker expression and low luminal oxygen profiles, it was unclear how capable these cells are of activating immune defenses when exposed to bacterial infections. To investigate this, we challenged the 3D intestine tissues with laboratory *E*. *coli* to determine if and how they respond to a well-known intestinal pathogen. Human intestines are constantly exposed to a vast number and diversity of bacteria[48]. To cope with the substantial microbial threats, the intestinal epithelium uses defense mechanisms which involve the activation of a number of microbial recognition and innate immune pathways, the secretion of diverse proinflammatory cytokines/chemokines and antimicrobial proteins to kill or prevent the growth of bacteria in infected tissues. In this study, we demonstrated that HIE-derived epithelia exhibit significant antibacterial responses, as evidenced by the increased expression of genes with important roles in pathogen recognition and the activation of immune responses, including microbial sensor genes, cytokines, inflammatory mediator genes, downstream signal transduction genes, and inflammasome signaling genes (Fig 5). Interestingly, many of these genes are activated in the intestinal tissues of IBD patients. For example, IBD patients have increased mRNA expression of Toll-like receptors, TLR2, TLR4 and TLR6, in the distal colon during colitis[49, 50]; CRP (C-reactive protein) is a clinical biomarker of IBD, as patients diagnosed with Crohn’s disease and ulcerative colitis have elevated CRP[51]; cytokines, such as IL-6, IL-12A, IL-12B, IL-1B and CXCL2, were upregulated in active IBD patients at diagnosis and during therapy[52–55]; the enhancement of both NOD1 and NOD2 mRNAs was detected in tissue biopsies from IBD patients[56]; the TNF serum level was significantly increased in IBD patients compared to healthy controls[57]; expression of SLPI mRNA are higher in patients with ulcerative colitis than in healthy controls or patients with Crohn’s disease[58]. Strikingly, multiple upregulated genes identified in the infected HIE-derived epithelium, including TLR6, CRP, CXCL12, SLPI, were not changed or only slightly upregulated in the infected hInEpiC-derived and cell line-derived epithelia. It has been reported that infection with *E*. *coli* triggers an immune response that may cause uncontrolled inflammation that occurs in Crohn’s disease and other types of IBD[59]. The results presented here suggested that the HIE-derived 3D intestinal epithelium not only replicates many *in vivo* characteristics of the human intestinal epithelium, but also closely reflects the human innate immune response to bacterial infection, which may permit the *in vitro* study of host-microbe-pathogen interplay and pathogenesis of IBD. It is well known that intestinal microbiota play an important role in the development of human IBD, including ulcerative colitis and Crohn's disease. In the future, we will introduce human fecal microbiota into the tissue to reproduce a model that mimics the intact human intestine to assess the impact of the microbiota on the pathogenesis of IBD. HIEs have been used for establishing models for new drug efficacy and toxicity testing [60, 61]. Additionally, they can be isolated from any compartments of the human small intestines, including jejunum, ileum, and duodenum. Therefore, the 3D nontransformed intestinal tissues derived from HIEs from different intestinal compartments will be beneficial for the study of drug specific toxicity, permeability and absorption sites in the intestinal tract. Furthermore, the small intestinal crypt-villus micro-environment appears to affect bacterial binding and colonization during infections [62]. In this respect, the accurate recreation of crypt-villus niche conditions on the luminal surface of the scaffolds to provide a more suitable micro-environment for the intestinal epithelial cell lining, should facilitate studies of bacterial colonization and enteric pathogen interactions.

## Conclusions

This study demonstrates the possibility of growing human intestinal enteroid-derived epithelial cells *in vitro* in a biocompatible 3D tubular silk scaffold system. Since HIEs are intestinal stem cells-derived, they can differentiate into all relevant intestinal epithelial cell types (enterocytes, Goblet cells, Paneth cells and enteroendocrine cells) required to recreate a physiologically relevant system. Importantly, HIEs are derived from native intestine tissues donated by individual patients, which allow this system to study patient-specific disease mechanisms and drug responses. Moreover, the 3D nontransformed intestinal epithelium tissue model closely mimics natural human infection. This promising feature will provide the basis for acute and chronic studies of interactions between the mammalian cells, bacterial infectious agents and the study of antibiotic resistance.

## Supporting information

S1 Table qRT‐PCR primer list. (DOCX)

S1 Fig Schematic illustrating the step-wise protocol of silk-based processing methods for the fabrication of 3D porous scaffolds for intestine engineering. (TIF)

S2 Fig Schematic of the experimental protocol for the cell isolation and seeding of enteroid-derived cells in porous silk scaffolds. (TIF)

S3 Fig SEM on the microvilli at the apical cell surface of different cell types. HIE-derived constructs possess higher packing density of microvilli and formed continuous brush borders across the cells than hInEpiC-derived and cell line-derived constructs.
(TIF)
