# Supplementary material for: In vitro enteroid-derived three-dimensional tissue model of human small intestinal epithelium with innate immune responses
Source: PLoS One. 2017 Nov 29;12(11):e0187880. doi: 10.1371/journal.pone.0187880 (PMC5706668; doi:10.1371/journal.pone.0187880)
Supplement: S1 Table — (DOCX) [file pone.0187880.s001.docx]

**S1 Table. qRT‐PCR primer list**

| **Gene products** | Forward | Reverse |
| --- | --- | --- |
| GAPDH | GAAGGTGAAGGTCGGAGTC | GAAGATGGTGATGGGATTTC |
| ZO-1 | CTGGTGAAATCCCGGAAAAATGA | TTGCTGCCAAACTATCTTGTGA |
| E-caherin | ATCGGTTGTTCAATGCGTCC | CCTTCAGGATTTGGTACATGACA |
| Villin | CGGAAAGCACCCGTATGGAG | CGTCCACCACGCCTACATAG |
| SI | TCCAGCTACTACTCGTGTGAC | CCCTCTGTTGGGAATTGTTCTG |
| Chga | ACTCCGAGGAGATGAACGGA | CTTGGAGAGCGAGGTCTTGG |
| Lysozyme | CGCTACTGGTGTAATGATGG | TTTGCACAAGCTACAGCATC |
| Muc-2 | TGCCTGGCCCTGTCTTTG | CAGCTCCAGCATGAGTGC |
| ALP | TACACGTCCATCCTGTACGG | CTCGCTCTCATTCACGTCTGG |
| Lgr-5 | GAGAAAGCATTTGTAGGCAAC | ATCTCCCAACAAACTGGATG |
