# Supplementary figures and images for: In vitro enteroid-derived three-dimensional tissue model of human small intestinal epithelium with innate immune responses
Source: PLoS One. 2017 Nov 29;12(11):e0187880. doi: 10.1371/journal.pone.0187880 (PMC5706668; doi:10.1371/journal.pone.0187880)

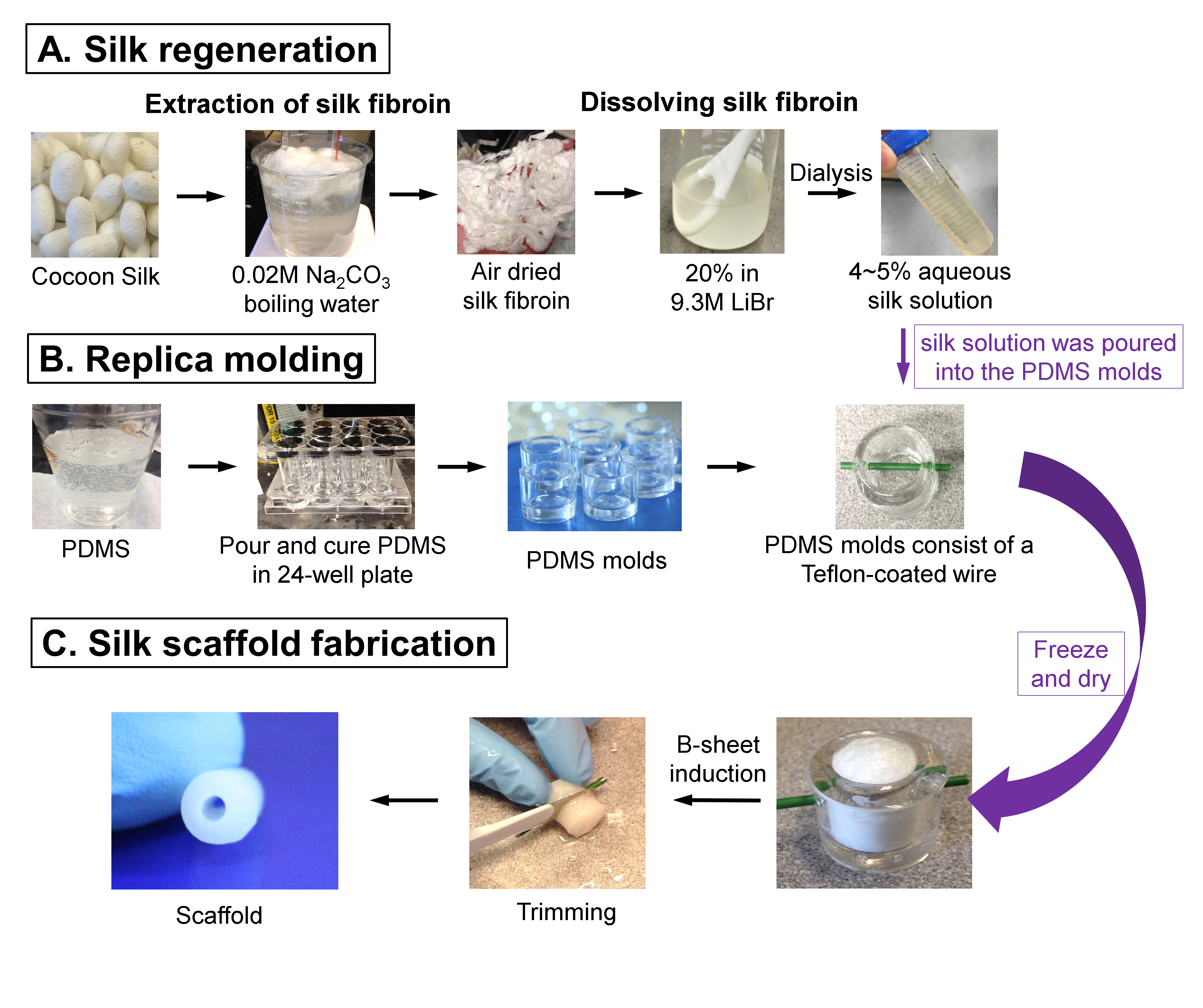

Supplement: S1 Fig — (TIF) [file pone.0187880.s002.tif]

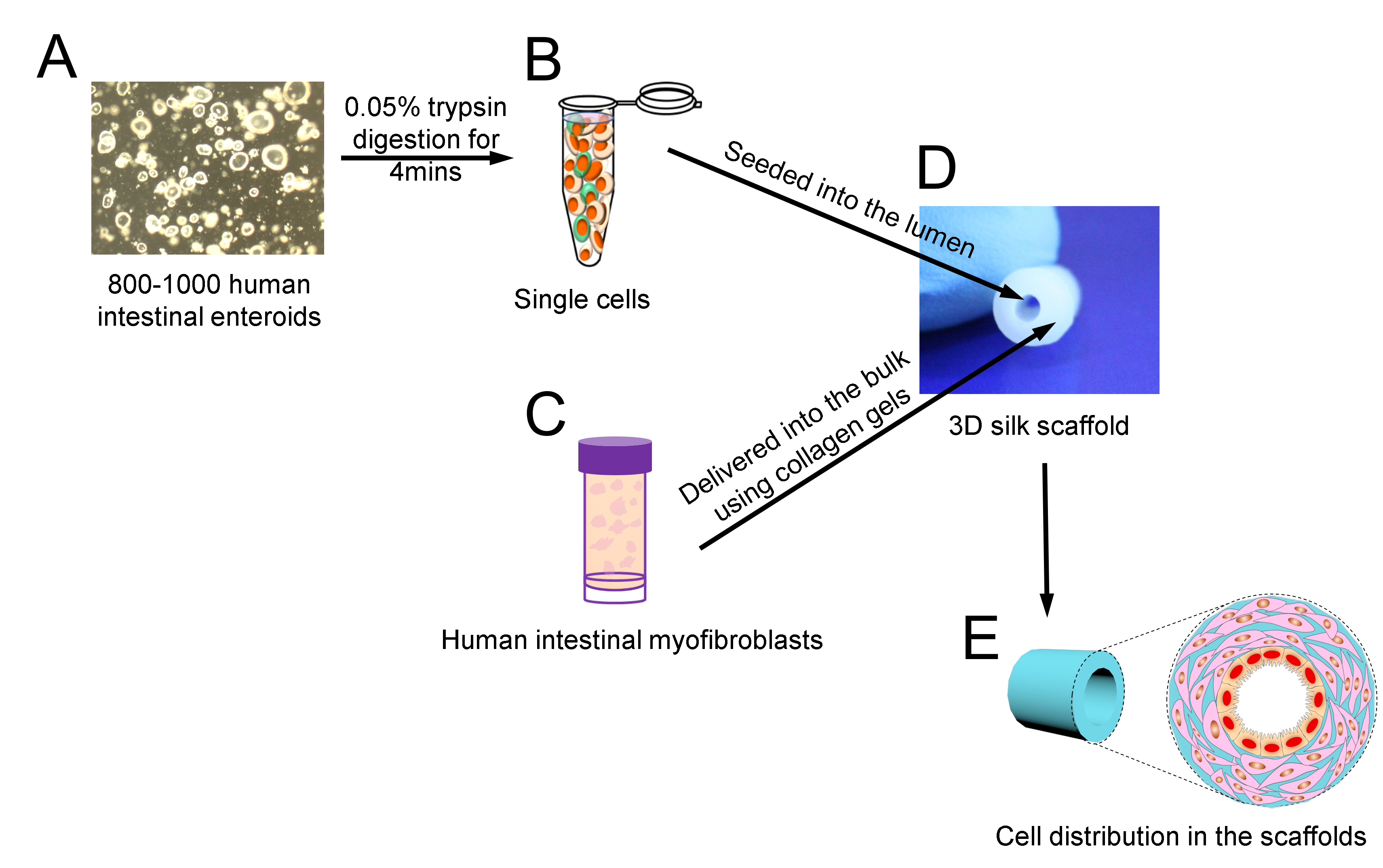

Supplement: S2 Fig — (TIF) [file pone.0187880.s003.tif]

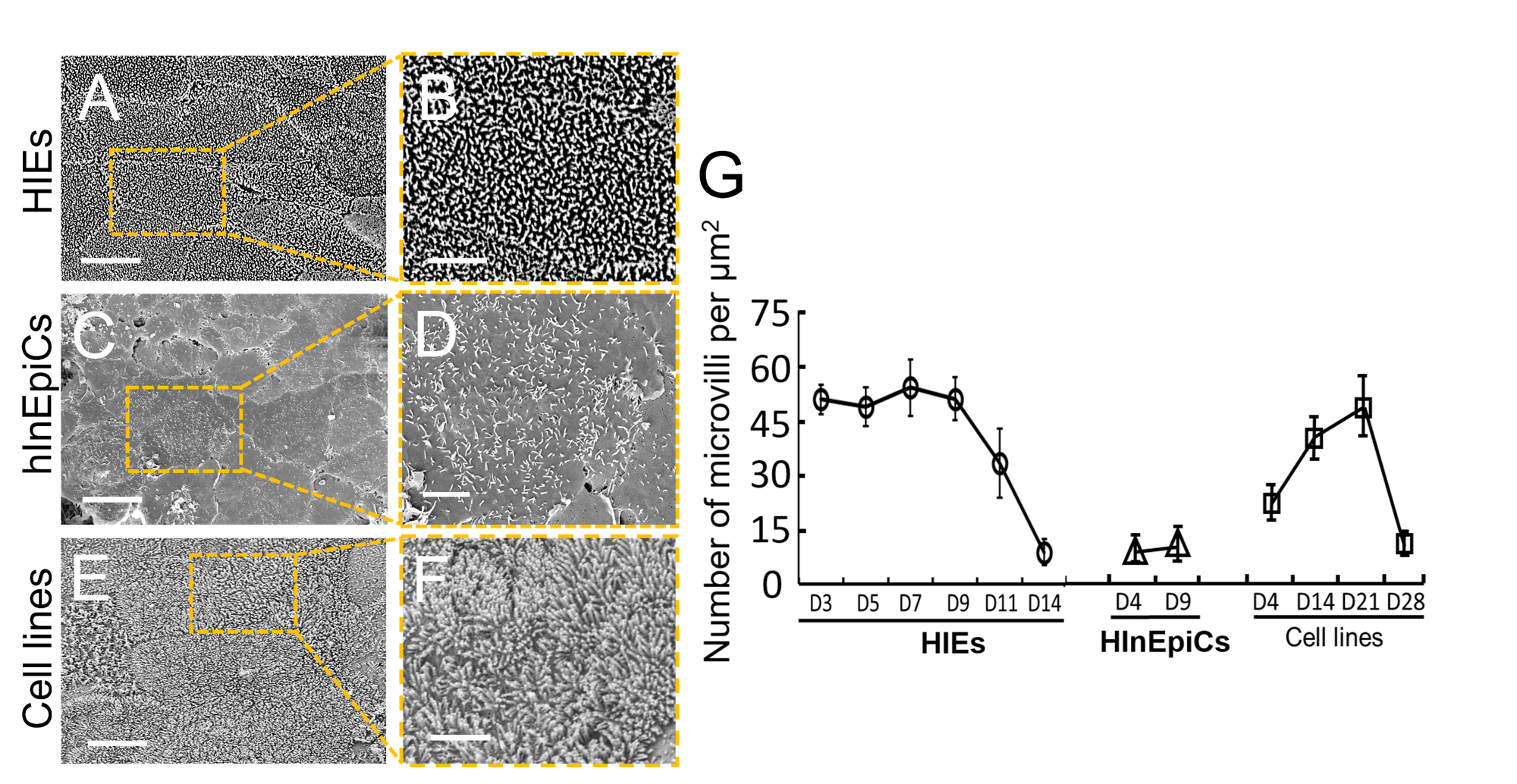

Supplement: S3 Fig — HIE-derived constructs possess higher packing density of microvilli and formed continuous brush borders across the cells than hInEpiC-derived and cell line-derived constructs. (TIF) [file pone.0187880.s004.tif]
